# LCZ696 ameliorates doxorubicin-induced cardiomyocyte toxicity in rats

**DOI:** 10.1038/s41598-022-09094-z

**Published:** 2022-03-23

**Authors:** Toru Miyoshi, Kazufumi Nakamura, Naofumi Amioka, Omer F. Hatipoglu, Tomoko Yonezawa, Yukihiro Saito, Masashi Yoshida, Satoshi Akagi, Hiroshi Ito

**Affiliations:** 1 Department of Cardiovascular Medicine, Okayama University Graduate School of Medicine, Dentistry and Pharmaceutical Sciences, Okayama University, 2-5-1 Shikata-cho, Kita-ku, Okayama, 700-8558 Japan; 2 Department of Pharmacology, Kindai University, Osaka, Japan; 3 Department of Molecular Biology and Biochemistry, Okayama University Graduate School of Medicine, Dentistry and Pharmaceutical Science, Okayama, Japan

**Keywords:** Cancer therapy, Heart failure

## Abstract

Doxorubicin (DOX)-based chemotherapy induces cardiotoxicity, which is considered the main bottleneck for its clinical application. In this study, we investigated the potential benefit of LCZ696, an angiotensin receptor–neprilysin inhibitor against DOX-induced cardiotoxicity in rats and H9c2 cells and determined whether the mechanism underlying any such effects involves its antioxidant activity. Male Sprague–Dawley rats were randomly separated into four groups, each consisting of 15 rats (DOX (1.5 mg/kg/day intraperitoneally for 10 days followed by non-treatment for 8 days); DOX + valsartan (31 mg/kg/day by gavage from day 1 to day 18); DOX + LCZ696 (68 mg/kg/day by gavage from day 1 to day 18); and control (saline intraperitoneally for 10 days). DOX-induced elevation of cardiac troponin T levels on day 18 was significantly reduced by LCZ696, but not valsartan. The DOX-induced increase in myocardial reactive oxygen species (ROS) levels determined using dihydroethidium was significantly ameliorated by LCZ696, but not valsartan, and was accompanied by the suppression of DOX-induced increase in p47phox. LCZ696 recovered the DOX-induced decrease in phosphorylation of adenosine monophosphate-activated protein kinase and increased the ratio of Bax and Bcl-2. In H9c2 cardiomyocytes, LCZ696 reduced DOX-induced mitochondrial ROS generation and improved cell viability more than valsartan. Our findings indicated that LCZ696 ameliorated DOX-induced cardiotoxicity in rat hearts in vivo and in vitro, possibly by mediating a decrease in oxidative stress.

## Introduction

Doxorubicin (DOX) is a highly effective chemotherapeutic anticancer agent; however, its use is restricted because of acute, subacute, and chronic cardiotoxicity, which eventually leads to severe heart failure (HF)^1^. Several experimental studies have demonstrated that the disruption of mitochondrial function and the generation of reactive oxygen species (ROS) play pivotal roles in DOX-induced cardiotoxicity, and that the administration of antioxidants protects cardiac tissues from DOX-induced oxidative stress injury^2–4^. Moreover, angiotensin converting enzyme (ACE) inhibitors/angiotensin receptor blockers (ARBs) ameliorated cardiotoxicity by reducing the oxidative stress and regulating the transforming growth factor-beta 1 pathway^5–8^. Furthermore, clinical trials demonstrated the potential benefit of ACE inhibitors/ARBs for the treatment of DOX-induced HF^9,10^.

LCZ696, an angiotensin receptor–neprilysin inhibitor comprises the molecular components of valsartan and sacubitril^11^. Sacubitril results in increased levels of peptide substrates of neprilysin, most notably the natriuretic peptides^12^. Clinical trials have shown that LCZ696 significantly decreased the overall mortality, HF symptoms, and hospitalizations for HF compared with enalapril, an ACE inhibitor^13^. Recently, we reported that LCZ696, but not valsartan, ameliorated isoproterenol-induced fibrosis in rats^14^. Other experimental studies showed that LCZ696 ameliorated oxidative stress in rodent models of obesity-related and diabetic cardiomyopathy, myocardial infarction, and pressure overload-induced cardiac remodeling^15–18^. Ge et al. reported that LCZ696 inhibits the nuclear transport of nuclear factor-κB and phosphorylation of c-Jun N-terminal kinase and p38 mitogen-activated kinase in H9c2 cardiomyocytes under high-glucose conditions^16^. However, data regarding the benefits of LCZ696 on DOX-induced cardiotoxicity is lacking. Herein, we investigated the potential benefit of LCZ696 against DOX-induced myocardial injury in rats (compared with valsartan), and whether its antioxidant activity is responsible for any such effects. Furthermore, we evaluated the protective effect of LCZ696 against oxidative stress and cytotoxicity in H9c2 cells (compared with valsartan).

## Results

### Effects of LCZ696 on DOX-induced changes in hemodynamic parameters and heart weight

Table 1 shows the change in heart rate, blood pressure, body weight, heart weight, heart weight to body weight ratio, fractional shortening, and ejection fraction in treated rats. During the experimental period, the change in heart rate over time did not differ among the four groups. Systolic blood pressure on day 11 in the DOX (rats that received DOX), DOX + VAL (rats that received DOX and valsartan), and DOX + LCZ (rats that received DOX and LCZ696) groups was significantly lower than that in the control (rats that received saline) group. On day 18, systolic blood pressure in the DOX + LCZ group was significantly lower than that in the control and DOX groups.

**Table 1:** Change in the heart rate, blood pressure, body weight, heart weight, heart weight to body weight ratio, fractional shortening, and ejection fraction in the treated rats.

Group	Control	DOX	DOX + VAL	DOX + LCZ
**Heart Rate, beats per minute**				
Day 0	339 ± 7	355 ± 8	335 ± 8	329 ± 9
Day 11	331 ± 8	336 ± 9	348 ± 9	330 ± 10
Day 18	318 ± 6	334 ± 7	341 ± 7	339 ± 8
**Systolic blood pressure, mmHg**				
Day 0	130 ± 1	129 ± 1	131 ± 1	129 ± 2
Day 11	126 ± 2	117 ± 2*	116 ± 2*	116 ± 2*
Day 18	139 ± 2	141 ± 2	135 ± 2	130 ± 2*^#^
**Body weight, g**				
Day 0	280.0 ± 3.8	281.6 ± 2.4	280.1 ± 2.9	282.7 ± 3.4
Day 11	338.3 ± 6.3	256.2 ± 6.6*	257.8 ± 5.7*	257.2 ± 6.4*
Day 18	365.9 ± 6.4	285.6 ± 6.0*	283.2 ± 5.9*	273.2 ± 9.3*
Heart weight (*), mg				
Day 18	965.8 ± 20.4	778.1 ± 20.2*	717.6 ± 12.7*	673.7 ± 23.2*^#^
**Heart weight to body weight ratio, mg/g**				
Day 18	2.64 ± 0.03	2.72 ± 0.04*	2.53 ± 0.04*^#^	2.47 ± 0.04*^#^
**Fractional shortening, %**				
Day 0	51.2 ± 1.0	48.9 ± 1.0	51.3 ± 1.1	51.2 ± 1.2
Day 11	51.2 ± 1.4	41.8 ± 1.5*	41.1 ± 1.6*	41.0 ± 1.7*
Day 18	50.4 ± 1.0	44.5 ± 1.5*	45.8 ± 1.5*	44.3 ± 1.2*
**Ejection fraction, %**				
Day 0	88.4 ± 0.7	86.4 ± 0.6	87.9 ± 0.6	88.1 ± 0.6
Day 11	88.2 ± 0.6	79.9 ± 1.3*	78.8 ± 1.9*	79.9 ± 1.3*
Day 18	87.6 ± 0.6	85.2 ± 0.9*	83.8 ± 0.9*	84.5 ± 0.8*

Data are expressed as the mean ± SEM. DOX, doxorubicin; VAL, valsartan; LCZ, LCZ696. n = 15 in each group. *p < 0.05 versus control and ^#^*p* < 0.05 versus DOX.

The heart weight to body weight ratio in the DOX group was significantly higher than that in the control group. However, the heart weight to body weight ratio in the DOX + VAL and DOX + LCZ groups was significantly lower than that in the control and DOX groups.

Left-ventricular (LV) function was assessed by echocardiography on days 0, 11, and 18. The fractional shortening and ejection fraction on day 11 in the DOX, DOX + VAL, and DOX + LCZ groups were significantly lower than those in the control group. On day 18, the same parameters remained significantly lower than those in the control group. DOX-induced LV dysfunction was not prevented by valsartan and LCZ696.

### Effects of LCZ696 on cardiac troponin T in DOX-treated rats

**Figure 1:**
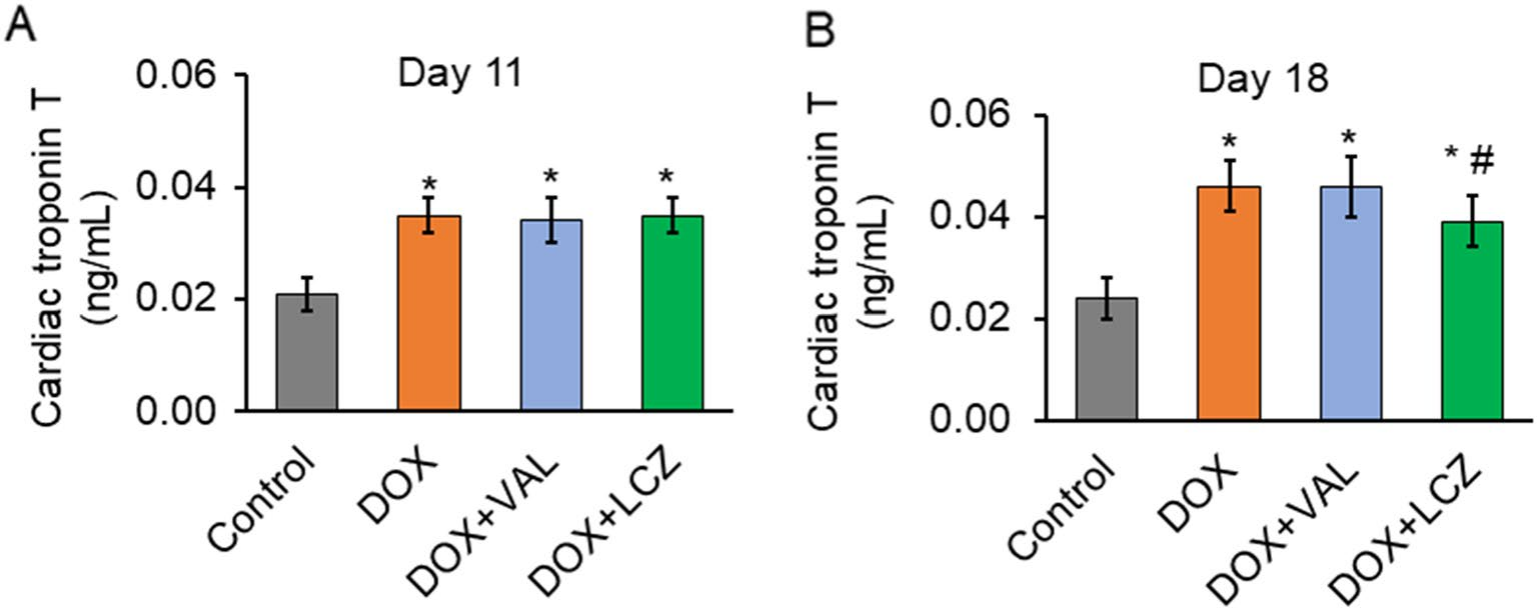
Effect of valsartan and LCZ696 on the serum cardiac troponin T levels in doxorubicin-treated rats. (**A**) On day 11, DOX significantly increased the serum cardiac troponin T level, which did not differ from that in the DOX + VAL and DOX + LCZ groups. n = 15 in each group. (**B**) On day 18, the increased serum cardiac troponin T level in the DOX group was significantly reduced in the DOX + LCZ group, but not in the DOX + VAL group. n = 10–15 in each group. Data are expressed as the mean ± standard error. *p < 0.05 versus control and ^#^*p* < 0.05 versus DOX. MDA, malondialdehyde; DOX, doxorubicin; VAL, valsartan; LCZ, LCZ696.

To assess the change in myocardial injury, the serum level of cardiac troponin T was measured. On day 11, it was increased in the DOX, DOX + VAL, and DOX + LCZ groups compared to that in the control group (Fig. 1A). On day 18, the same parameter was increased in the DOX group and significantly reduced in the DOX + LCZ group, but not in the DOX + VAL group (Fig. 1B).

### Effects of LCZ696 on DOX-induced oxidative stress in rat

**Figure 2:**
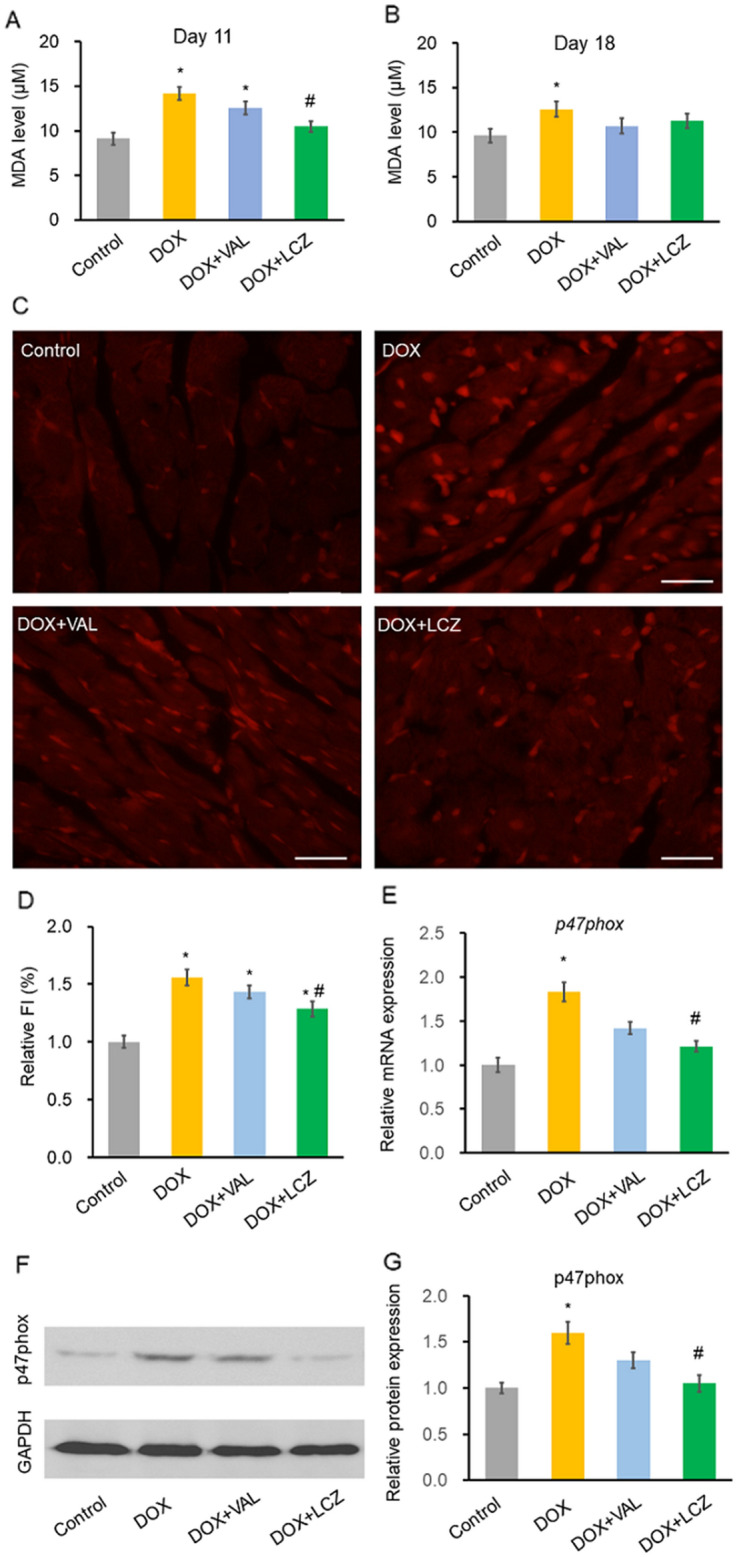
Effect of valsartan and LCZ696 on serum malondialdehyde (MDA) levels and oxidative stress in rat hearts treated with doxorubicin. (**A**) On day 11, DOX significantly increased the serum MDA level which was significantly reduced by LCZ, but not VAL. n = 15 in each group. (**B**) On day 18, DOX significantly increased the serum MDA level, which was not reduced by LCZ and VAL. n = 10–15 in each group. (**C**) Representative DHE staining on day 18 in each group. (**D**) Oxidative stress in the cardiac tissue was measured based on the fluorescence intensity of DHE. LCZ, but not VAL, significantly reduced the DOX-induced oxidative stress. Five random fields from three random samples per group were re-evaluated for the analysis. (**E**) Real-time PCR to check for the expression of p47phox day 18. DOX significantly increased p47phox mRNA expression. This increase was significantly reduced by LCZ, but not VAL. Data are presented relative to the control group. n = 10–15 in each group. (**F**) Representative Western blot images of the expression of p47phox in the cardiac tissue on day 18. (**G**) Protein expression of p47phox as measured by western blotting. DOX significantly increased the level of p47phox which was significantly reduced by LCZ, but not VAL. Data are presented relative to the control group. Five random samples per group were re-evaluated for the analysis. Data are expressed as the mean ± standard error. Scale bars, 50 μm. *p < 0.05 versus control and ^#^*p* < 0.05 versus DOX. MDA, malondialdehyde; DOX, doxorubicin; VAL, valsartan; LCZ, LCZ696; DHE, dihydroethidium; FI, fluorescence intensity; TNF, tumor necrosis factor.

We measured the MDA level to test whether pre-treatment with LCZ696 ameliorated DOX-induced oxidative stress in rats. On day 11, the serum MDA level was significantly reduced in the DOX + LCZ group compared to that in the DOX group (Fig. 2A). On day 18, these levels did not differ among the DOX, DOX + VAL, and DOX + LCZ groups (Fig. 2B).

To evaluate ROS production in cardiac tissue, dihydroethidium (DHE) staining of the frozen LV section of rats was performed. The fluorescence intensity of DHE, which was increased in the DOX group, was significantly reduced in the DOX + LCZ group, but not in the DOX + VAL group (Fig. 2C,D).

We investigated the expression of p47phox, a key subunit of nicotinamide adenine dinucleotide phosphate oxidase that plays a pivotal role in inducing oxidative stress^19^. On day 18, real-time polymerase chain reaction (PCR) and western blotting showed that the mRNA and protein expression of p47phox was increased in the DOX group (Fig. 2E,F,G). These increases in expression were significantly reduced by LCZ696, but not valsartan.

### Effects of LCZ696 on AMPK and Bcl-2 family in the cardiac tissue

**Figure 3:**
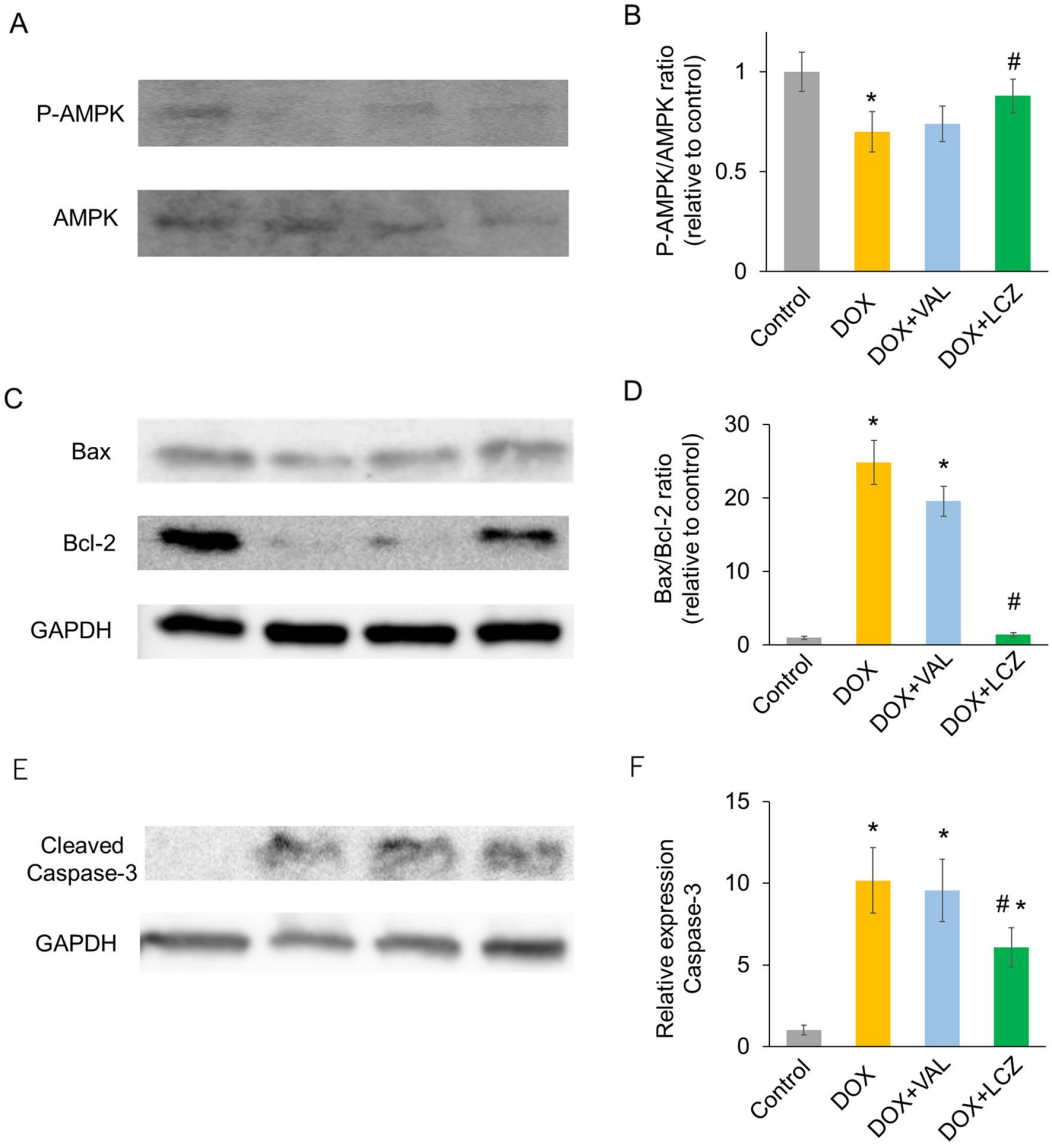
Effect of valsartan and LCZ696 on activation of AMPK and the expression of Bax, Bcl-2, and cleaved caspase3 in rat hearts treated with doxorubicin. (**A**) Representative western blot images of the expression of phosphorylated- and total-AMPK in the cardiac tissue on day 18. (**B**) The ratio of p-AMPK/AMPK as measured by western blotting. DOX significantly decreased the ratio of p-AMPK/AMPK, which LCZ significantly increased, but not VAL. (**C**) Representative western blot images of the expression of Bax and Bcl-2 in the cardiac tissue on day 18. (**D**) The ratio of p-AMPK/AMPK as measured by western blotting. DOX significantly increased the ratio of Bax/Bcl-2, which LCZ significantly reduced, but not VAL. (**E**) Representative western blot images of the expression of cleaved caspase-3 in the cardiac tissue on day 18. (**F**) Protein expression of cleaved caspase-3 as measured by western blotting. DOX significantly increased the level of caspase-3, which LCZ significantly reduced, but not VAL. Data are expressed as the mean ± standard error. *p < 0.05 versus control and ^#^*p* < 0.05 versus p-AMPK; phosphorylated adenosine monophosphate-activated protein kinase; DOX, doxorubicin; VAL, valsartan; LCZ, LCZ696.

Previous studies have demonstrated that DOX inhibits AMPK activation^20^. The phosphorylated AMPK level was decreased in the DOX group compared to that in the control group (Fig. 3A,B). This alteration of phosphorylated AMPK induced by DOX was recovered significantly by the LCZ696 group but not by valsartan.

Proteins of the Bcl-2 family play critical roles in regulating apoptosis. Bax and Bcl-2 are key players in this family. The expression of Bax did not differ among the control, DOX, DOX + VAL, and DOX + LCZ696 groups (Fig. 3C). However, the expression of Bcl-2 decreased in the DOX group and was recovered by LCZ696. The DOX group's Bax/Bcl-2 ratio increase was also significantly reduced by LCZ696, but not valsartan (Fig. 3D). In addition, we evaluated the effect of LCZ696 on the activation of caspase-3 (Fig. 3E,F). The level of cleaved caspase-3 protein in the DOX group was significantly higher than that in the control group. This alteration of cleaved caspase-3 protein expression induced by DOX was reversed by LCZ696, but not valsartan.

### Effects of LCZ696 on DOX-induced morphological changes in rat myocardium

**Figure 4:**
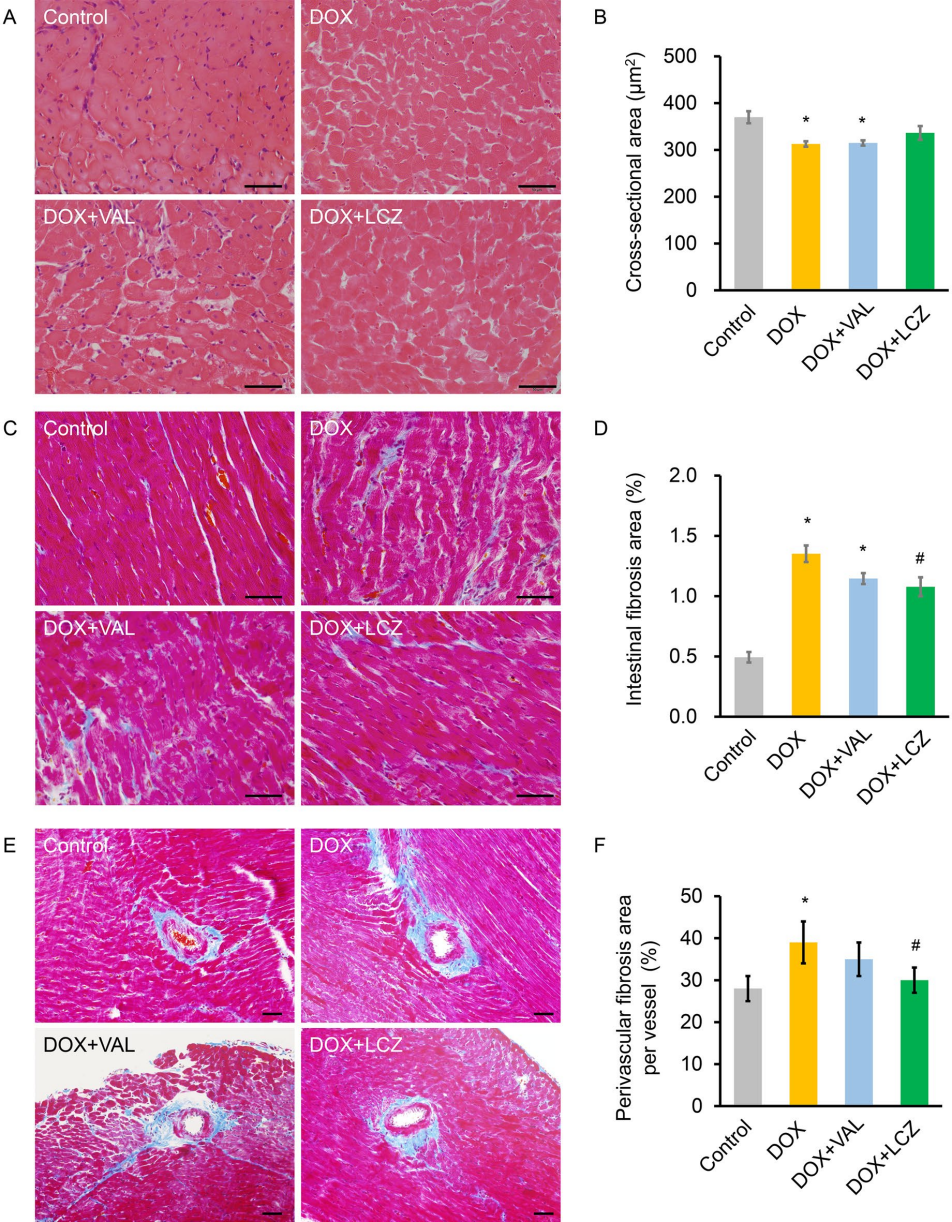
Effect of valsartan and LCZ696 on morphological changes in rat heart treated with doxorubicin. (**A**) Representative hematoxylin and eosin and Masson’s trichrome staining on day 18 in each group. (**B**) DOX significantly decreased the cross-sectional area of cardiomyocytes compared to the control group, whereas the increase in the cross-sectional area of cardiomyocytes by VAL and LCZ was not significant. (**C**) Representative Masson’s trichrome staining of intestinal fibrosis on day 18 in each group. (**D**) The percentage of intestinal fibrotic area was significantly increased by DOX. LCZ, but not VAL significantly reduced the percentage of intestinal fibrosis induced by DOX. (**E**) Representative Masson’s trichrome staining of perivascular fibrosis on day 18 in each group. (**F**) The percentage of perivascular fibrotic area was significantly increased upon DOX treatment. LCZ, but not valsartan, significantly reduced the DOX-induced percentage perivascular fibrotic area. Data are expressed as the mean ± standard error. Scale bars, 50 μm. Five random fields from three random samples per group were evaluated for the analysis. *p < 0.05 versus control and ^#^*p* < 0.05 versus DOX. DOX, doxorubicin; VAL, valsartan; LCZ, LCZ696.

To microscopically investigate the effect of LCZ696 on DOX-induced change in rat myocardium, we used hematoxylin and eosin (HE) and Masson’s trichrome staining. HE staining showed that the cross-sectional area of cardiomyocytes was significantly lesser in the DOX and DOX + VAL groups, but not in the DOX + LCZ group, when compared to that in the control group (Fig. 4A,B). Furthermore, with respect to the effect of LCZ696 on cardiac fibrosis in rats, Masson’s trichrome staining showed increased intestinal fibrosis in the DOX group, and significantly decreased intestinal fibrosis in the DOX + LCZ group, but not in the DOX + VAL group (Fig. 4C,D). The increase in perivascular fibrosis in the DOX group was significantly reduced in the DOX + LCZ group, but not in the DOX + LCZ group (Fig. 4E and 4F).

### Expression of collagen type I alpha 1 (COL1A1), tumor necrosis factor-alpha (TNFa), and atrial natriuretic peptide (ANP) mRNA in the cardiac tissue

**Figure 5:**
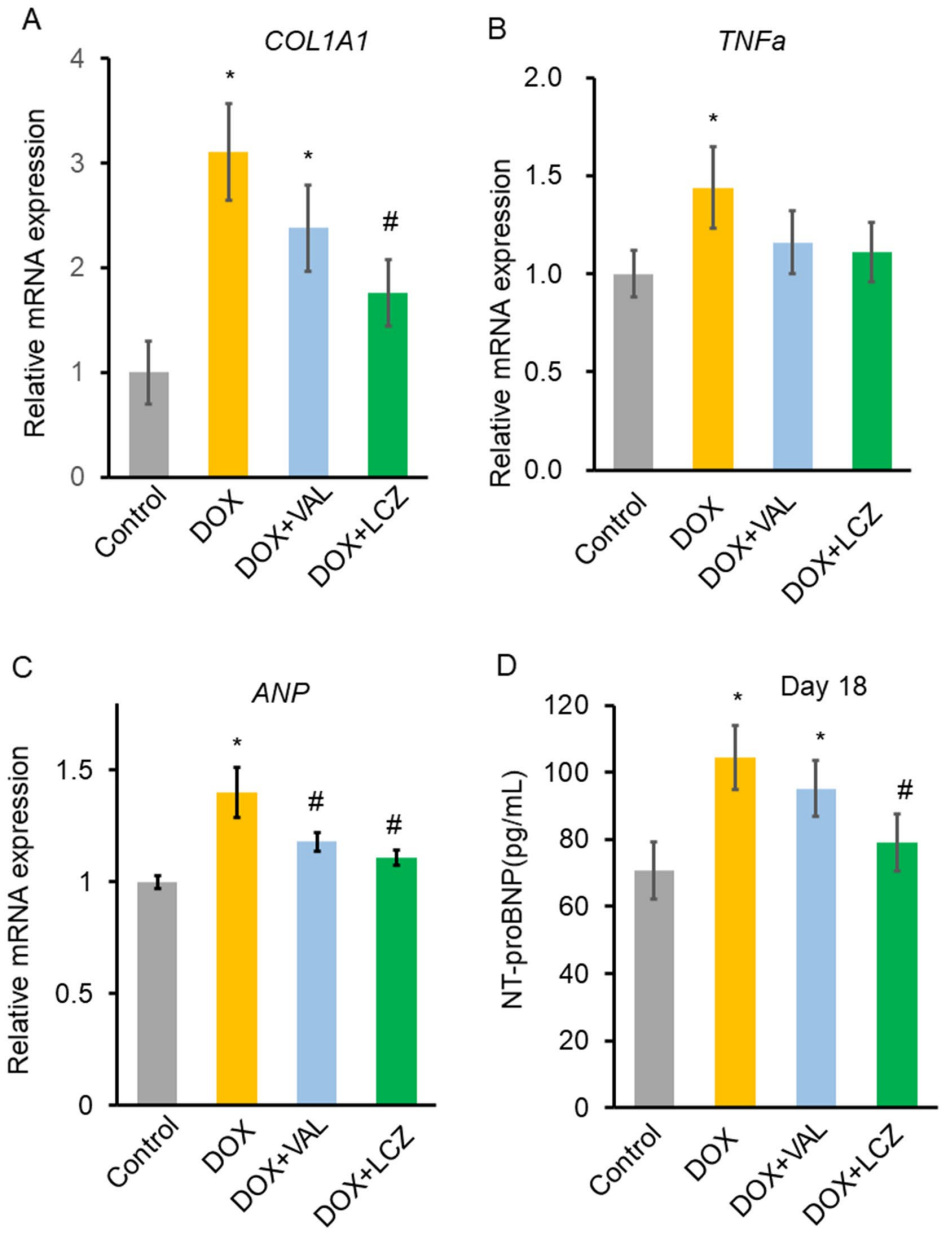
Effect of valsartan and LCZ696 on the mRNA expression of collagen, tumor necrosis factor-alpha (TNFa), and atrial natriuretic peptide (ANP) in the rat heart, and on serum N-terminal pro-brain natriuretic peptide (NT-proBNP) levels in DOX-treated rats. (**A**) Real-time PCR to check for the expression of *COL1A1* mRNA on day 18. The DOX-induced increase in the mRNA levels of *COL1A1* was significantly suppressed by LCZ, but not VAL. n = 10–15 in each group. (**B**) Real-time PCR analysis of *TNFa* mRNA on day 18. The DOX-induced increase in the mRNA levels of *TNFa* was not reduced by VAL or LCZ significantly. n = 10–15 in each group. (**C**) Real-time PCR to check for the expression of *ANP* mRNA on day 18. The DOX-induced increase in the mRNA levels of *ANP* was significantly suppressed by VAL or LCZ. n = 10–15 in each group. (**D**) Serum NT-proBNP level was significantly increased upon DOX treatment. LCZ, but not VAL, significantly reduced the serum NT-proBNP levels that were increased upon DOX treatment. n = 10–15 in each group. Data are expressed as the mean ± standard error. *p < 0.05 versus control and ^#^*p* < 0.05 versus DOX. DOX, doxorubicin; VAL, valsartan; LCZ, LCZ696; COL1A1, collagen type I alpha 1; TNFa, tumor necrosis factor-alpha; ANP, atrial natriuretic peptide; NT-proBNP, N-terminal pro-brain natriuretic peptide.

Real-time PCR was performed to evaluate the mRNA expression of collagen. On day 18, the mRNA levels of *COL1A1* in the DOX and DOX + VAL groups were significantly increased compared to those in the control group (Fig. 5A). The mRNA level of *COL1A1* in the DOX + LCZ, but not in the DOX + VAL, was significantly lower than that in the DOX group.

Because TNFa plays an important role in DOX-induced cardiotoxicity^21,22^, the expression of *TNFa* mRNA was evaluated using real-time PCR (Fig. 5B). On day 18, it was significantly higher in the DOX group than that in the control; however, the increased expression of *TNFa* in response to DOX treatment was not significantly reduced upon treatment with valsartan or LCZ696.

We also analyzed the expression of *ANP* mRNA using real-time PCR (Fig. 5C). On day 18, it was significantly higher in the DOX group than that in the control. The increased expression of *ANP* in response to DOX was significantly reduced upon treatment with valsartan or LCZ696.

### Measurements of serum N-terminal pro-brain natriuretic peptide (NT-proBNP) levels

Figure 5D shows the serum NT-proBNP level on day 18. The NT-proBNP level was increased in the DOX group and was significantly decreased in the DOX + LCZ group, but not the DOX + VAL group (control, 70.7 ± 8.4 pg/mL; DOX, 104.4 ± 9.6 pg/mL; DOX + VAL, 95.2 ± 8.5 pg/mL; and DOX + LCZ, 79.1 ± 8.4 pg/mL).

### Effects of LCZ696 on DOX-induced mitochondrial oxidative stress in H9c2 cells

**Figure 6:**
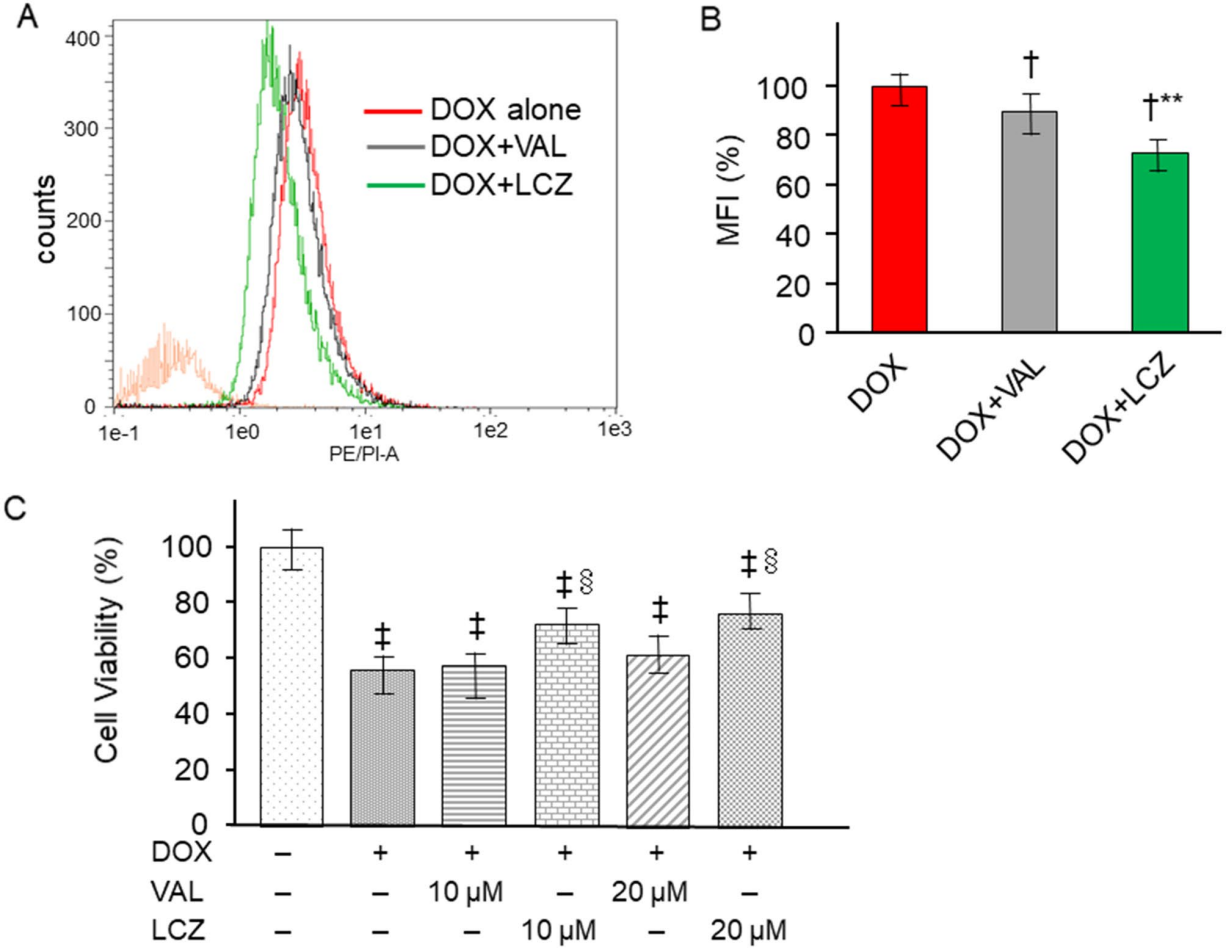
Effect of LCZ696 on doxorubicin-induced mitochondrial oxidative stress and cytotoxicity in H9c2 cells. (**A**) Cells were stained for superoxide using MitoSOX, a fluorescence indicator of mitochondrial superoxide, and were assayed via flow cytometry. Cells were pre-treated with 20 μM VAL and LCZ for 12 h, and then treated with 5 µM of doxorubicin for 6 h. (**B**) The mean fluorescence intensity was measured through flow cytometry in individual cells and plotted. LCZ effectively attenuated DOX-induced superoxide generation. Data are expressed as the mean ± standard error. n = 3 in each group. ^†^*p* < 0.01 versus DOX and **p < 0.05 versus DOX + VAL. (**C**) Cell viability was measured with MTS assay and expressed as the percentage of control. DOX markedly decreased cell viability. Pre-treatment with 10 μM and 20 μM LCZ ameliorated cytotoxicity, whereas pre-treatment with VAL did not inhibit DOX-induced cytotoxicity. Each point represents the mean ± standard error. n = 5 in each group. ^‡^*p* < 0.05 versus control and ^§^*p* < 0.05 versus DOX treatment alone. DOX, doxorubicin; VAL, valsartan; LCZ, LCZ696; MFI, mean fluorescence intensity.

We investigated the effect of valsartan and LCZ696 on DOX-induced mitochondrial ROS level in H9c2 cells. Mitochondrial superoxide production was measured via fluorescence-activated cell sorting-based quantification of fluorescent MitoSOX Red. As shown in Fig. 6A, H9c2 cells displayed marked mitochondrial ROS production after 6 h of treatment with 5 μM DOX, whereas mitochondrial ROS production was clearly inhibited in the cells pre-treated with LCZ696. The mean fluorescence of intensity increased above that of the control and DOX-treated cells. However, pre-treatment with valsartan attenuated the increased fluorescence, and pre-treatment with LCZ696 further inhibited the increased fluorescence (Fig. 6B).

### Effects of LCZ696 on DOX-induced cytotoxicity in H9c2 cells

To analyze the effect of LCZ696 on DOX-induced cytotoxicity, cell viability was measured using MTS assay after exposing H9c2 cells to DOX. As shown in Fig. 6C, cell viability was markedly decreased after 24 h of treatment with 5 μM DOX. Pre-treatment with 10 μM and 20 μM LCZ696 for 12 h attenuated the decreased cell viability, whereas pre-treatment with 10 μM and 20 μM valsartan did not inhibit DOX-induced cytotoxicity.

## Discussion

Herein, we investigated the prophylactic effects of LCZ696 against DOX-induced cardiotoxicity and the underlying potential mechanisms. Our results showed that LCZ696 treatment ameliorated DOX-induced oxidative stress and cardiac fibrosis in rats. However, DOX-induced cardiac dysfunction was not prevented by LCZ696 in this model.

The study of hemodynamic parameters in the DOX group showed a significant decrease in fractional shortening and ejection fraction which were not prevented by valsartan and LCZ696. Heart weight and heart weight to body weight ratio were reduced by DOX and a significant decrease in systolic blood pressure during treatment with DOX was observed, consistent with previous reports^5,23^. These experiments were performed to investigate the early pathophysiological mechanisms of DOX-induced cardiotoxicity. Boutagy et al. recently reported that LCZ696 attenuated the DOX-induced reduction of LV systolic function in rats receiving DOX over 3 weeks, which contradicts our findings^24^. However, we obtained insufficient evidence for the efficacy of LCZ696, possibly because of the short treatment duration. With respect to the change in systolic blood pressure, its reduction on day 11 was similar among the DOX, DOX + VAL, and DOX + LCZ groups, probably due to DOX-induced LV dysfunction. However, the reduction in systolic blood pressure on day 18 in the DOX + LCZ group, but not in the DOX VAL groups, remained significant compared to that in the control and DOX groups. An explanation is that sacubitril in LCZ696 elevates the levels of circulating natriuretic peptides such as ANP, BNP, and C-type natriuretic peptide, which induce vasodilation^25^.

This study demonstrated that treatment with LCZ696 reduced DOX-induced oxidative stress both in rats and H9c2 cells, and that this effect of LCZ696 was stronger than that of valsartan. The inhibition of ARB suppresses oxidative stress in spontaneously hypertensive rats^26^. In addition, in cardiomyocytes, natriuretic peptides play a role in the development of the anti-oxidative stress response that is induced upon the inhibition of NADPH oxidases^27^. Consistent with previous data, this study showed that the DOX-induced upregulation of 47phox was reduced by LCZ696. Thus, the additive antioxidant effects of angiotensin blockade and increased natriuretic peptides may contribute to the greater effect of LCZ696. Mitochondria are a major source of ROS during HF^28^. Xia et al. reported that LCZ696 alleviated the increase in dynamin-related protein 1, a fission protein of mitochondria, in DOX-induced cardiotoxicity^29^. This study suggests that a decrease in ROS production may indicate mitochondrial dysfunction in the DOX-induced cardiotoxicity model.

Mitochondrial impairment, increased apoptosis, dysregulated autophagy, and increased fibrosis have also been shown to play crucial roles in doxorubicin cardiotoxicity. AMPK30 links these cellular processes. Several studies have shown that cardiac AMPK is inhibited by DOX^20,30^. In this study, LCZ696 significantly increased the expression of phosphorylated AMPK. The effect of LCZ696 on AMPK activation may be involved in ameliorating DOX-induced cardiotoxicity in rat hearts.

Proteins of the Bcl-2 family play critical roles in the regulation of apoptosis^31^. Bax and Bcl-2 are the key players in this family, and the ratio of Bax/Bcl-2 is critical for the induction of apoptosis. The increased ratio of BAX/Bcl-2 leads to the activation of caspase-3^32^. We found that LCZ696 decreased the increased ratio of Bax/Bcl-2 by DOX and an increased in cleaved caspase -3. These data suggest that the anti-apoptotic effect contributed to the alleviation of DOX-induced cardiotoxicity.

Our study also demonstrated that LCZ696 ameliorated cardiac fibrosis. An antifibrotic effect of LCZ696 has been reported in rodent models of HF characterized by pressure-overload cardiac hypertrophy^33^, myocardial infarction^34^, chronic kidney disease^35^, isoproterenol-induced cardiotoxicity^14^, diabetic cardiomyopathy^36^, DOX-induced dilated cardiomyopathy^29^, and obesity-related diastolic dysfunction^15^. Although the precise mechanisms underlying the protective effect of LCZ696 in preventing DOX-induced cardiac fibrosis remain unclear, we previously reported that the mRNA level of transforming growth factor = beta in isoproterenol-treated left ventricle was suppressed in the LCZ696 group compared with that of the control groups^14^. Neprilysin inhibition increases circulating levels of natriuretic peptides, which would increase the generation of cyclic guanosine monophosphate^11^. The increased cyclic guanosine monophosphate is a potent inhibitor of collagen production through transforming growth factor-beta in fibroblasts in vitro^25^. Consistent with our findings, in the sub-analysis of the PARADIGM-HF trial, the effects of sacubitril/valsartan on biomarkers of extracellular matrix in heart failure with reduced ejection fraction were evaluated, and sacubitril/valsartan was found to reduce profibrotic signaling^37^.

This study had a few limitations. First, it evaluated the effect of LCZ696 on the early pathophysiological change in DOX-induced cardiomyopathy. Further study is needed to clarify the benefit of LCZ696 on chronic DOX-induced cardiotoxicity. Second, although the differential effects between LCZ696 and valsartan may be due to neprilysin blockade, the neprilysin substrates responsible cannot be determined. Neprilysin has several peptide substrates including bradykinin, substance P, adrenomedullin, angiotensin I and II, and endothelin-1^38^. In this study, we did not evaluate the specific effect of sacubitril on cardiotoxicity after treatment with LCZ696. Therefore, it is necessary to determine the exact mechanisms contributing to the beneficial effects of LCZ696.

In summary, our data demonstrated that LCZ696 protected the rat hearts from DOX-induced cardiotoxicity in vivo and in vitro. This effect may be mediated via decreased oxidative stress. Our results suggest that LCZ696 can be potentially used to treat DOX-induced cardiotoxicity. Potential clinical benefits should be investigated in large prospective clinical studies in the future.

## Methods

### Ethical approval

This investigation conforms to the *Guide for the Care and Use of Laboratory Animals* published by the US National Institutes of Health (NIH Publication No. 85–23, revised 1985). All procedures were performed in accordance with the institutional guidelines for animal research and approved by the Animal Care and Use Committee of Okayama University (OKU-2017589). This study was carried out in compliance with the ARRIVE guidelines.

### Rat model of DOX-induced cardiomyopathy

Sixty male Sprague–Dawley rats weighing 255–305 g at the beginning of the experiment were purchased from Japan SLC (Shizuoka, Japan). They were housed at an animal facility in a 12-h light/dark cycle and provided with standard rat chow and water ad libitum. They were randomly allocated to four groups (n = 15) as follows: (1) Control: rats received 2 mL/kg/day saline intraperitoneally from day 1 to day 10 followed by non-treatment for 8 days. (2) DOX: rats received 1.5 mg/kg/day DOX (Wako, Tokyo, Japan) intraperitoneally from day 1 to day 10 followed by non-treatment for 8 days, as previously described^5^. A total dose of 15 mg/kg DOX was administered over the 10-day period. (3) DOX + VAL: rats received 1.5 mg/kg/day DOX intraperitoneally from day 1 to day 10 followed by non-treatment for 8 days and 31 mg/kg/day valsartan from day 1 to day 18 orally. (4) DOX + LCZ: rats received 1.5 mg/kg/day DOX intraperitoneally from day 1 to day 10 followed by non-treatment for 8 days and 68 mg/kg/day LCZ696 from day 1 to day 18 orally. On day 18, the rats were anesthetized by administering 60 mg/kg sodium thiopental intraperitoneally and euthanized. Valsartan was purchased from Sigma-Aldrich (St. Louis, MO, USA). LCZ696 for animal experiments was supplied by Novartis Pharma K.K. (Basel, Switzerland) and included molecular moieties of valsartan and sacubitril at a 1:1 ratio. In this study, doses of 68 mg/kg and 31 mg/kg, respectively were selected for LCZ696 and valsartan because several experimental studies using rat models have employed these doses^14,35,36,39,40^.

### Blood pressure and pulse rate measurement

Systolic blood pressure and pulse rate were measured at 0, 11 and 18 days using tail-cuff plethysmography (MK-2000, Muromachi, Tokyo, Japan)^41^. An average of three measurements was used.

### Echocardiography

Transthoracic echocardiography was performed using a 10-MHz phased array transducer (Aplio ver. 6.0; Toshiba, Tokyo, Japan) under 2% isoflurane. The papillary muscles were visualized with M-mode echocardiography using a parasternal short-axis view. The LV internal diameter at end-diastole and end-systole are measured during diastole and systole. Fractional shortening was then calculated according to the formula FS = [(LV internal diameter at end-diastole) − (LV internal diameter at end-systole)/LV internal diameter at end-diastole] × 100. Ejection fraction was calculated using end-systolic and end-diastolic volumes according to Teichholz’s formula^37^.

### Measurements of cardiac troponin T

Serum cardiac troponin T was measured using an enzyme-linked immunosorbent assay kit (RK03996, ABclonal, Woburn, MA, USA) according to the protocol. Briefly, the samples and standard substances were added into 96-well plate and incubated for 60 min at 37 °C, cultured with biotin-conjugated antibodies for 60 min, and later seeded with streptavidin–peroxidase complex for 30 min before adding tetramethylbenzidine substrate. Finally, the optical density values at 450 nm were detected using a microplate reader (Bio-Rad Laboratories, Hercules, CA) for calculating the quantity.

### Determination of thiobarbituric acid reactive substances (TBARS)

Serum oxidative stress in the subjects were evaluated using TBARS assay kit (Cayman Chemical, Ann Arbor, MI, USA) according to the manufacturer’s recommendations. Malondialdehyde (MDA)–thiobarbituric acid (TBA) adduct formed by the reaction of MDA and TBA at high temperature (90–100 °C) was measured colorimetrically at 530–540 nm using a microplate reader (FlexStation 3, Molecular Devices, CA, USA). The MDA concentration is expressed in μM.

### Histology

The LV cardiac tissues taken for histological analysis were fixed with 4% paraformaldehyde prepared in phosphate-buffered saline, embedded in paraffin, and cut into 5-µm-thick sections. Subsequently, these sections were stained with HE for evaluating the cross-sectional areas of the cardiomyocytes and Masson’s trichrome for evaluating the percentage of fibrosis compared to the total area. The cardiomyocyte cross-sectional areas and fibrotic area were examined using a BZ-700 microscope equipped with a BZ analyzer (Keyence, Osaka, Japan). Intestinal fibrosis area (%) and perivascular fibrosis area (%) were calculated using the following formulas: intestinal fibrosis = collagen area/total area × 100 and perivascular fibrosis area (%) = area occupied by the collagen/total area of the vessel section × 100. The percentage of the fibrotic area was analyzed using WinROOF Version 5.7 (MITANI Corporation, Fukui, Japan)^42^.

### Detection of superoxide production by fluorescence histology

Superoxide production in frozen LV tissue (5 μm sections) was evaluated by DHE (5 mmol/L, 30 min, 37℃) staining. DHE was purchased from Sigma-Aldrich (St. Louis, MO, USA). DHE fluorescence was detected using an Olympus DP70 camera (Tokyo, Japan) mounted on a fluorescent microscope (Olympus IX71, Tokyo, Japan) with an excitation wavelength of 488 nm and an emission wavelength of 568 nm.

### Real-time PCR

Total RNA was extracted using the TRIzol reagent (Invitrogen, Carlsbad, CA) according to the manufacturer’s protocol. RNA was quantified using Implen’s Nanophotometer (Munich, Germany). cDNA was synthesized using the Prime Script RT reagent kit (Takara). Real-time qPCR was performed using the StepOnePlus Real-Time PCR System (Applied Biosystems)^43^. All mRNA-specific labeled primers were purchased from Applied Biosystems and the expression of the following genes was investigated: *Col1a1* (Rn01523309_m), *Tnfa* (Rn01525859_g1), and *Anp* (Rn00664637_g1). *Gapdh* (Rn01775763_g1) was used as an internal control, and the data were analyzed using the 2^−ΔΔCt^ method^43^.

### Western blotting

Protein samples from the heart of five randomly selected rats in each group were prepared using a BEAD crusher (µT-12, Taitec, Koshigaya, Japan). Tissue lysates were prepared using a radioimmunoprecipitation buffer supplemented with 2 mmol/L phenylmethylsulfonyl fluoride, 1 mmol/L sodium orthovanadate, and 10 mmol/L sodium fluoride (sc-24948, Santa Cruz, Dallas, Texas, USA) and 20 µg of lysates was subjected to sodium dodecyl sulphate–polyacrylamide gel electrophoresis. Goat anti-NCF1/p47phox antibody (#SAB2500674, Sigma-Aldrich, St. Louis, MO, USA), rabbit anti-AMPK antibody (#2603, Cell Signaling Technology), rabbit anti-phosphorylated AMPK antibody (#2535, Cell Signaling Technology), rabbit anti-Bax antibody (#2772, Cell Signaling Technology), rabbit anti-Bcl-2 antibody (#D038-3, MBL, Tokyo, Japan), rabbit anti-cleaved caspase-3 antibody (#9664, Cell Signaling Technology), and rabbit anti-GAPDH antibody (#2118, Cell Signaling Technology) were used. All primary antibodies were used at a dilution of 1:1000. The secondary antibodies were horseradish peroxidase-conjugated anti-rabbit IgG (NA934, GE Healthcare Bio-Sciences, Buckinghamshire, England) and horseradish peroxidase-conjugated anti-goat IgG (sc-2354, Santa Cruz). were used. All primary antibodies were used at a dilution of 1:1000. The secondary antibody was horseradish peroxidase-conjugated anti-rabbit IgG (NA934, GE Healthcare Bio-Sciences, Buckinghamshire, England). Chemiluminescence was detected using a chemiluminescence system (ECL Plus, GE Healthcare Bio-Sciences)^14^.

### Measurements of NT-proBNP

Serum NT-proBNP was measured using an enzyme-linked immunosorbent assay (MBS704791, MyBioSource, San Diego, CA, USA).

### H9c2 cell culture

H9c2 cells derived from the rat myocardium were obtained from the American Type Culture Collection (ATCC, Manassas, VA, USA). H9c2 cells were cultured in high glucose Dulbecco’s modified Eagle’s medium, supplemented with 10% fetal bovine serum, 1% penicillin/streptomycin, and 2 mM L-glutamine. Dulbecco’s modified Eagle’s medium, fetal bovine serum, and penicillin/streptomycin were purchased from Gibco (Grand Island, NY, USA). Cells were maintained in a humidified incubator (37 °C, 5% CO_2_) and were always used at less than 80% confluence. For cell culture experiments, LCZ696 was purchased from Cayman Chemical (Ann Arbor, MI, USA).

### Measurement of superoxide production in H9C2 cells

H9c2 cells (1 × 10^5^/well) were seeded in 12-well plates one day before the treatment. Cells were pre-treated with 10 μM and 20 μM each of valsartan and LCZ696 for 12 h and then treated with 5 µM of DOX for 6 h. Superoxide production was measured with a final concentration of 5 μM MitoSOX Red (Invitrogen, Grand Island, NY, USA) according to the manufacturer’s recommendation for 30 min at 37 °C in the dark. After treatment, cells were collected after washing three times with phosphate-buffered saline. Mean fluorescence intensity was detected by flow cytometry using MACSQuant Analyzer (Miltenyi Biotec, Bergisch Gladbach, Germany) and analyzed with the MACSQuantify 2.5 software.

### MTS cell viability assay

Cell viability was measured using MTS cell viability assay (Promega Corporation, Madison, WI, USA). H9c2 cells (1 × 10^4^/well) were seeded in 96-well plates one day before the treatment. Cells were pre-treated with 10 μM and 20 μM each of valsartan and LCZ696 for 12 h, and then treated with 5 µM of DOX for 24 h. Cell viability was determined by adding the MTS cell proliferation solution to each well and incubating the cells at 37 °C for 2 h. A FlexStation-3 plate reader (Molecular Devices, CA, USA) was used to measure the cell viability at 490 nm.

### Statistical analysis

Data are expressed as the mean ± standard error. Comparisons between multiple groups were done by the one‐way analysis of variance followed by Bonferroni post hoc test for data with a normal distribution or Kruskal–Wallis test with Dunn’s post hoc test for non-normal distribution data. A *p* value < 0.05 was considered statistically significant. Data were analyzed using SPSS for Windows (ver. 25; SPSS Inc., Chicago, IL, USA).

## Supplementary Information

Supplementary Information.

## Data Availability

The datasets generated during the present study are available from the corresponding author on reasonable request.
