# Supplementary material for: LCZ696 ameliorates doxorubicin-induced cardiomyocyte toxicity in rats
Source: Sci Rep. 2022 Mar 23;12:4930. doi: 10.1038/s41598-022-09094-z (PMC8943022; doi:10.1038/s41598-022-09094-z)
Supplement: Supplementary file 1 — Supplementary Information. [file 41598_2022_9094_MOESM1_ESM.pdf]

LCZ696 ameliorates doxorubicin-induced cardiomyocyte toxicity in rats

Toru Miyoshi[1*], Kazufumi Nakamura[1], Naofumi Amioka[1], Omer F. Hatipoglu[2], Tomoko Yonezawa[3], Yukihiro Saito[1], Masashi Yoshida[1], Satoshi Akagi[1], Hiroshi Ito[1]

1. Department of Cardiovascular Medicine, Okayama University Graduate School of Medicine, Dentistry and Pharmaceutical Sciences, Okayama, Japan
2. Department of Pharmacology, Kindai University, Osaka, Japan.
3. Department of Molecular Biology and Biochemistry, Okayama University Graduate School of Medicine, Dentistry and Pharmaceutical Science, Okayama, Japan

Correspondence: Toru Miyoshi, MD, miyoshit@cc.okayama-u.ac.jp

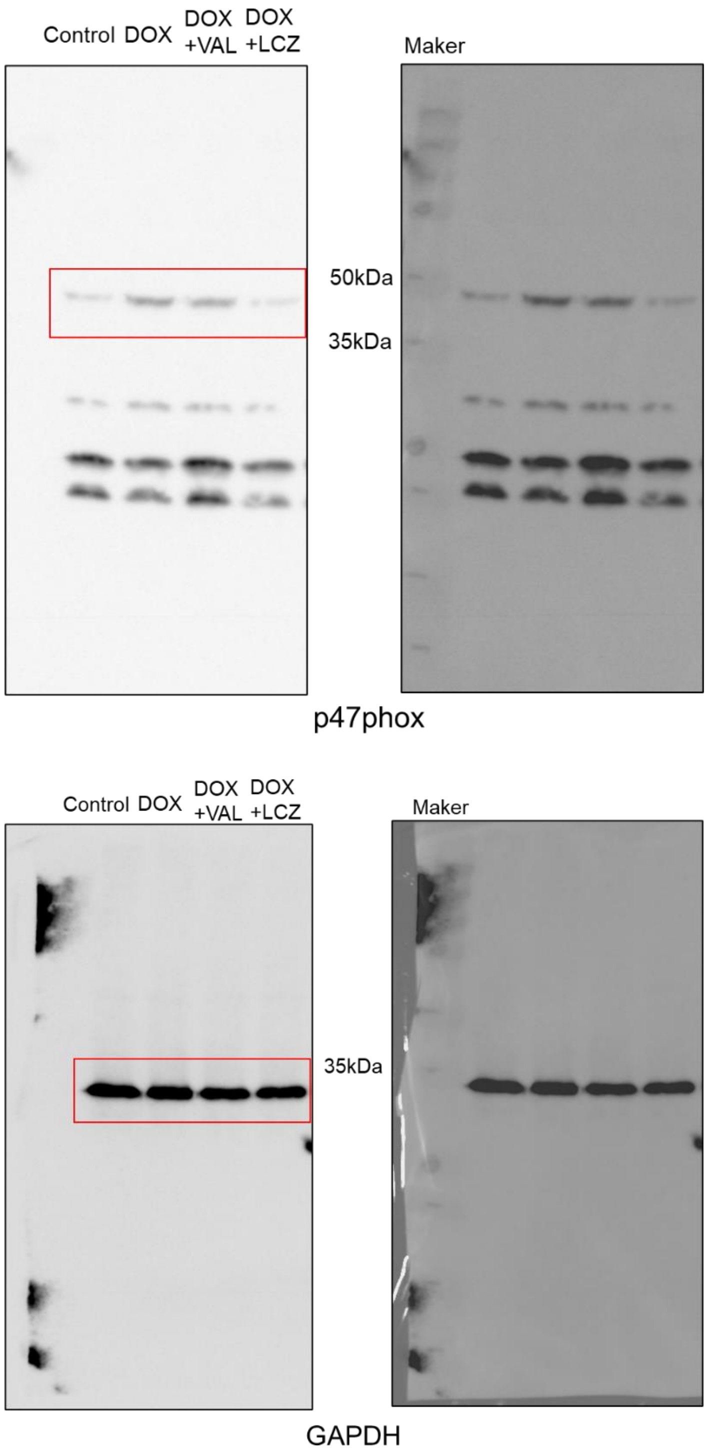

Supplementary Figure S1. Original western blot images used in Figure 2F.

The individual area bounded by solid red lines are cropped and shown in the corresponding figures. The blot on the right was superimposed with an image of the protein ladder to show the molecular weight of the bands. Protein band intensity was quantified using ImageJ software v1.8.0_172 (National Institute of Health, Maryland, USA).

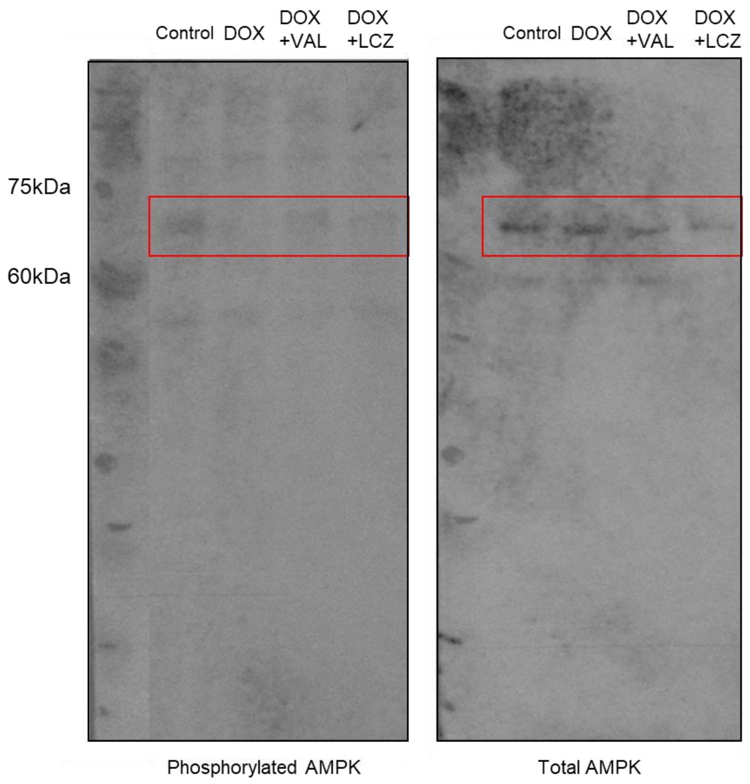

Supplementary Figure S2. Original western blot images used in Figure 3A.

The individual area bounded by solid red lines are cropped and shown in the corresponding figures. The blot on the right was superimposed with an image of the protein ladder to show the molecular weight of the bands. Protein band intensity was quantified using ImageJ software v1.8.0_172 (National Institute of Health, Maryland, USA).

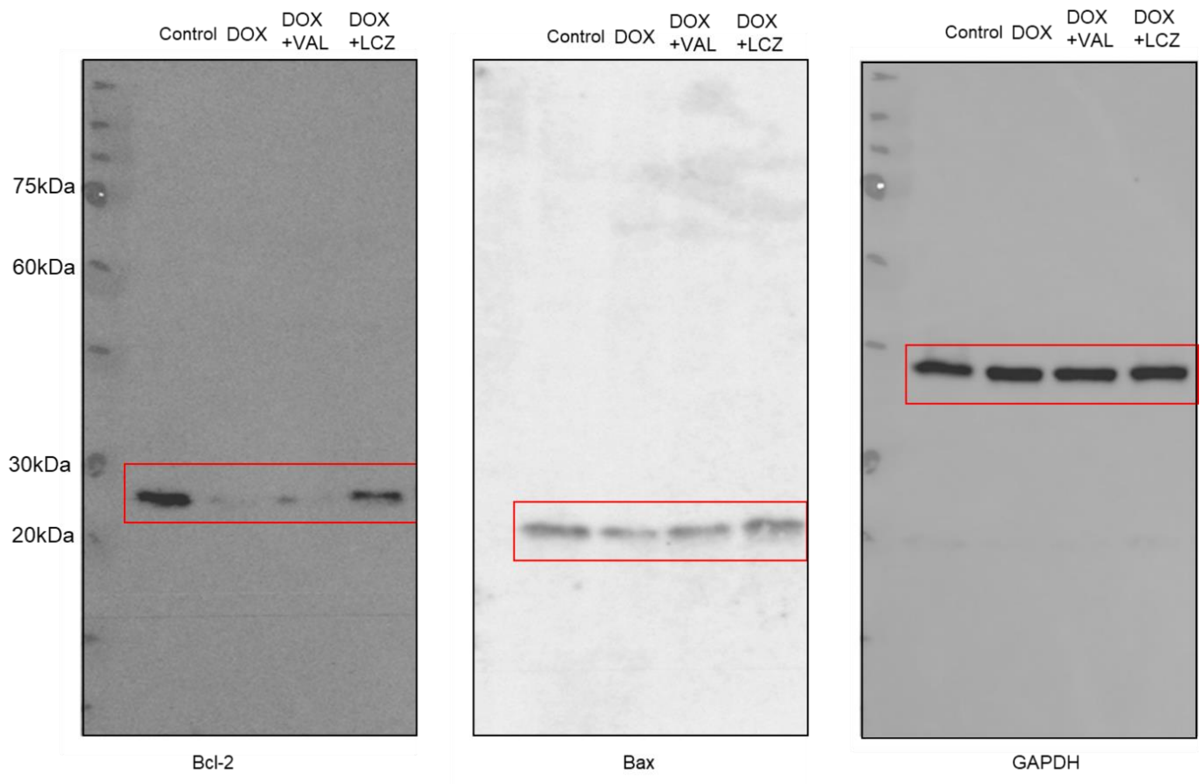

Supplementary Figure S3. Original western blot images used in Figure 3C.

The individual area bounded by solid red lines are cropped and shown in the corresponding figures. The blot on the right was superimposed with an image of the protein ladder to show the molecular weight of the bands. Protein band intensity was quantified using ImageJ software v1.8.0_172 (National Institute of Health, Maryland, USA).

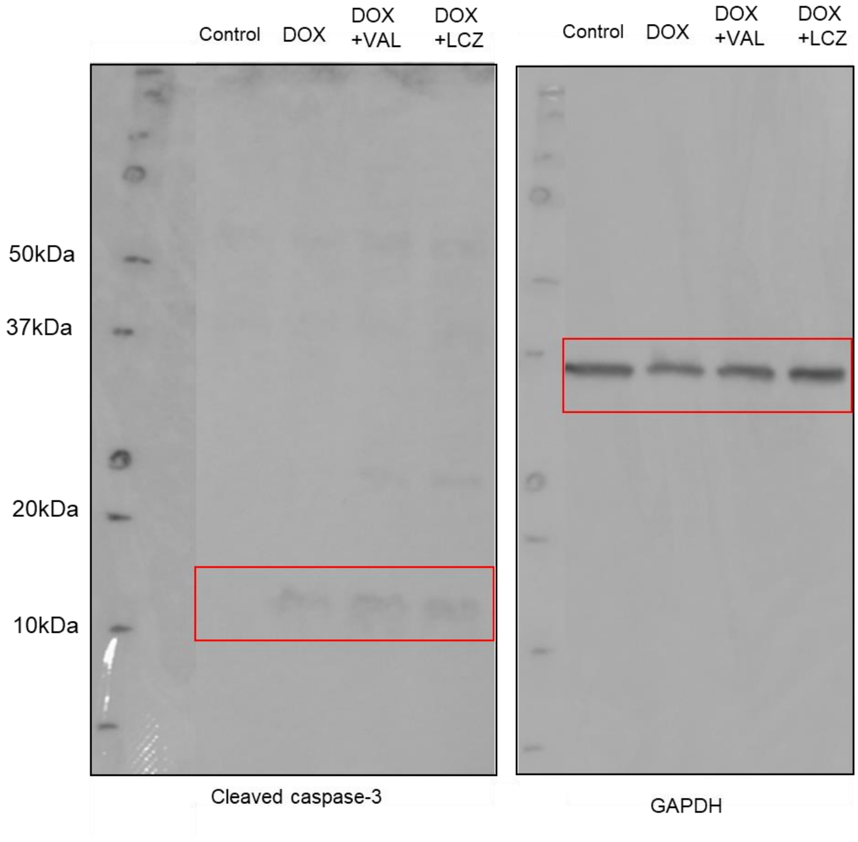

Supplementary Figure S4. Original western blot images used in Figure 3E.

The individual area bounded by solid red lines are cropped and shown in the corresponding figures. The blot on the right was superimposed with an image of the protein ladder to show the molecular weight of the bands. Protein band intensity was quantified using ImageJ software v1.8.0_172 (National Institute of Health, Maryland, USA).
